# Risk Factors for Low Back Pain in Youth Inline Hockey Players During the Season—A Prospective Cohort Research

**DOI:** 10.3390/children11121517

**Published:** 2024-12-14

**Authors:** Antonio Cejudo, Víctor Jesús Moreno-Alcaraz, Pilar Sainz de Baranda

**Affiliations:** Department of Physical Activity and Sport, Faculty of Sport Sciences, CEIR Campus Mare Nostrum (CMN), University of Murcia, 30720 Murcia, Spain; victorjm@um.es (V.J.M.-A.); psainzdebaranda@um.es (P.S.d.B.)

**Keywords:** hockey, injury prevention, back pain, risk factors, ROM-SPORT battery, muscle extensibility

## Abstract

Background: Low back pain is one of the most common musculoskeletal complaints in team sports. A screening test can help understand why injuries occur and predict who is at risk for non-contact low back pain. The objectives of the research were (1) to create models using logistic regression analysis of limited lower-extremity ranges of motion to prospectively identify potential factors for in-season non-contact non-contact low back pain and (2) to determine a training threshold (cut-off) for the identified factors in inline hockey players. Methods: A prospective cohort research was performed with 49 male inline hockey players aged 8 to 15 years. Data were collected regarding age, body composition, sports antecedents, competition level, and lower-limb ranges of motion (ROM-SPORT battery, *n* = 11 tests). A prospective measurement of non-contact low back pain was performed after 1 year (outcome) by asking the players supervised by the medical staff team (questionnaire). Results: Sixteen players (32.7%) experienced non-contact low back pain during the 1-year surveillance period. The model showed a significant relationship (χ^2^(39) = 43.939; *p* < 0.001) between the low back pain and the predictor variable hip flexion with the knee extended range of motion (OR = 3.850 [large]; 95% CI = 1.293 to 11.463; *p* = 0.015). The Bayesian Information Criteria and the Akaike Information Criteria for model fit were 56.885 and 37.967, respectively. The training threshold for hip flexion with the knee extended of ≤67° was set, which has an acceptable (area under the curve ≥ 94.1%) discriminatory ability for the development of non-contact low back pain for the screening test. Conclusions: Hamstring extensibility at 67° or less, as determined by hip flexion with knee extension, is a predictor of non-contact low back pain in youth inline hockey players.

## 1. Introduction

Inline hockey (IH) is a version of hockey played on a hard, smooth surface, with players using inline skates to move around and hockey sticks to drive, pass to a co-player, or hit a plastic puck into an opponent’s goal. The characteristics of the sport’s play, such as predisposition to injury [1,2,3]. Participation in a very physically and technically demanding hockey game (high-speed skating, rapid changes of direction, unintentional contact with players, contact with the boards, the stick, or the puck, and falls) leads to a notable increase in injury risk. Previous studies have shown a high total injury rate (from 139 to 300 injuries per 1000 athlete exposures) and incidence of sports injury IH (304.9 injuries per 1000 h of play) [1,2,3]. Previous epidemiological studies have reported that the frequency and severity of injuries in youth athletes increase and peak in adolescence [4,5], when skeletal structures grow rapidly and unevenly, leading to alterations in both physical performance and motor control/function [6,7]. The head/neck, shoulder, knee, ankle, and spine are the most frequently injured body regions in IH players [1,2,3].

In the spine, low back pain (LBP) is a musculoskeletal complaint that is already relatively common in athletes of team sports such as football [8,9], basketball [10], handball [11,12], ice hockey [12,13], and field hockey [8,13,14]. The retrospective (in the last 12 months) incidence of non-contact LBP in international IH players was reported to be 70% [15]. Sagittal misalignments of the spine have been associated with non-contact LBP in athletes [16,17,18,19]. Of 74 male hockey players (aged 8 to 15 years), 1.4% had lumbar hyperlordosis, and 9.5% had hypolordosis or rectification in a relaxed standing position; 68.9% had lumbar hyperkyphosis in a slump sitting position, and 44.6% had lumbar hyperkyphosis in maximum flexion of the trunk [20]. A high external and total hip rotation range of motion (ROM) was identified as a physical factor associated with non-contact LBP in 20 international male and female hockey players with an average age of 22.50 years [15]. In this regard, limited ROM due to muscle tightness is prevalent in IH players; limited ROM has a prevalence between 20% and 100% in the lower-extremity (hip and knee) except hip abduction ROM in 20 male and female international IH players with an average age of 22.50 years [15]. In 74 male youth IH players (aged 8 to 15 years), the prevalence of limited ROM was found to be between 27% and 100% in ankle dorsiflexion with the knee flexed, knee flexion, hip extension, hip rotation, hip flexion with the knee extended, hip adduction and hip abduction with the hip flexed [21].

It is generally agreed that injury such as LBP is a multifactorial phenomenon in which several factors of different natures (e.g., sociodemographic and sports characteristics, psychological constructs, neuromechanical variables) cooperate in a non-linear manner (complex link) and may affect the probability (i.e., risk) of occurrence (or non-occurrence) in an athlete (i.e., IH player). For this reason, athletic trainers and strength and conditioning coaches need to design a specific training multi-component (strength, flexibility, mechanics, and stability) program that optimizes the factors of different natures (e.g., personal characteristics, psychological constructs, neuro-mechanical parameters including flexibility [15] and posture [18,19,20]) of IH players to reduce the risk of sports injuries [22,23,24,25] or non-contact LBP [26,27,28]. Specifically, these professionals want to know which lower-limb movements have the greatest impact on the occurrence of non-contact LBP, and they want to know the reference values that determine training targets for muscle flexibility that result in a low risk of non-contact LBP. In this context, to the authors’ knowledge, only non-contact LBP was associated with ROM of external and total hip rotation with values of 56° and 93° or less, respectively, in 20 international senior IH players [15]. As far as the authors are aware, previous research has not analyzed the factors associated with non-contact LBP in youth players who participate in IH.

Most studies to detect potential factors linked with non-contact LBP mainly use a prospective cohort design and binary logistic regression and ROC analyses [29,30,31,32,33]. In this way, the cause-effect association is established in IH players who have a limited ROM of the lower limbs and subsequently develop a non-contact LBP during the sports season (or prospective surveillance period) [34]. The results of this research could be a good example of the first step towards a screening ROM test and help to understand why injuries occur and predict who is at risk of non-contact LBP [34]. On the other hand, retrospective follow-up is not recommended because it is difficult for athletes to remember exactly when the injury occurred (selection and recall bias) and because it is impossible to determine whether the injury is the cause or the consequence [35].

In order to better know the role of ROM in non-contact LBP, prospective cohort research was designed (1) to create models using logistic regression analysis of limited lower-extremity ROMs to prospectively identify potential factors for in-season non-contact LBP and (2) to determine a training threshold (cut-off) for the identified factors in 8 to 15-year inline hockey players. It was hypothesized that low hip rotation, extension, and flexion ROMs, as well as high mass body, are risk factors for the development of non-contact LBP in 8- to 15-year IH players.

## 2. Materials and Methods

### 2.1. Ethics Committee Approval Statement

The research was conducted in accordance with the ethical standards of the Declaration of Helsinki. The experimental procedures were submitted and approved by the Institutional Review Board of the University of Murcia (Spain) (Reg. Code 1702/2017, 5 May 2016). The TRIPOD guidelines were used in the development of the research [36]. The TRIPOD checklist can be found in the Supplementary File S1.

### 2.2. Research Design

A prospective cohort research was developed. The non-contact LBP sustained during training and competition was recorded over a one-year surveillance period after the in-season phase assessment session. Prior to the research, the directors of the National Technical Plan, the technical teams, and the parents/guardians of the participants were informed in detail about the research procedure and the purpose of the research and gave their written consent to participate in the research.

**Figure 1:**
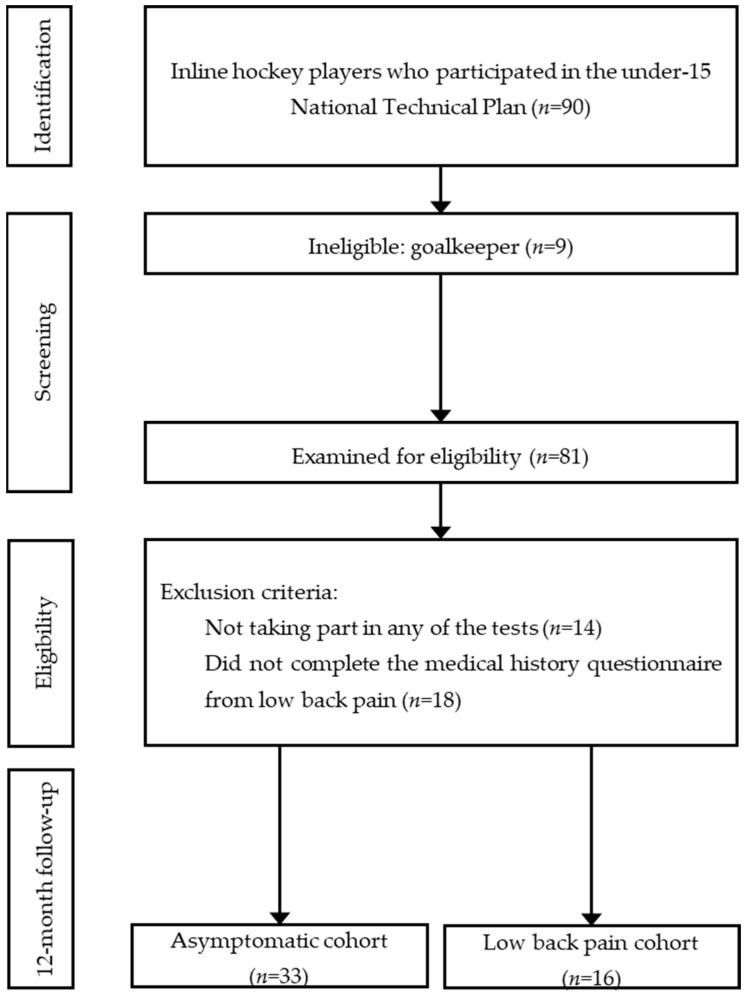
Flow chart of the follow-up cohort research.

A convenience sample of 90 participants (Figure 1) was selected from the National Technical Plan published by the Royal Spanish Skating Federation (aged 8 to 15 years). Goalkeepers did not participate in this research (*n* = 9) as flexibility is specific to the tactical role of the game [37,38] and also has different body composition, morphological, and performance profiles [39,40]. In addition, 32 IH players were excluded because they had only partially participated in the tests or had not completed the health history survey from non-contact LBP. Finally, forty-nine IH players demonstrated the predefined exclusion and inclusion requirements.

A day before the start of the National Technical Plan, two weeks before the National Technical Plan check-in, an introductory session of the ROM tests was conducted with the players. As part of the National Technical Plan, the independent variables were measured (hip, knee, and ankle ROMs) using a pre-set assessment battery (ROM-SPORT); information on confounding variables such as body composition, age, IH antecedents and competition level of the participants was also recorded. Measurement of these variables was conducted prior to daily IH training in a pavilion under standard 24 °C conditions. Players completed an aerobic and stretching (dynamic flexibility) warm-up before the ROM test. The tests were carried out by two evaluators with more than 16 years of experience in musculoskeletal assessment (PhD in Sports Science and Athletic Trainers). Both experts were blinded to the survey used and did not know whether the player had suffered from non-contact LBP. The tests were recorded simultaneously at random (due to time constraints). Each test was repeated 3 times. The mean value of the two closest measurements was used for the statistical analysis. The preferred limb was detected during the performance of two unilateral stabilization tasks (ball kick and initial pushing leg in a frontal movement).

Finally, after 1 year, a prospective measurement of non-contact LBP was completed (outcome) by interviewing the participants under the supervision of medical staff (survey). In this way, the participants were exposed to the training/competition of the autonomous community and the National Technical Plan for 1 year (exposure).

### 2.3. Participants

Forty-nine male IH players participating in the National Technical Plan and aged between 8 and 15 years took part in this investigation. The players were 11.9 ± 1.5 years old, had a body weight of 49.8 ± 10.1 kg, a height of 153.2 ± 10.2 cm, and a BMI of 21.2 ± 3.1 kg/m^2^. There were 24 players in the Benjamin category (U-10), 30 players in the Alevín category (U-12), and 20 players in the Infantil/Cadete category (U-15). The players routinely trained 3 times a week on non-consecutive days (2.88 ± 0.33 h per week) and played 1-competitive match per week (usually on weekends) during the season in the previous 1 year. The IH practice experience was 3.1 ± 1.6 years, 9.9 ± 0.8 months of training per year, and a training load of 4.2 ± 1.3 training hours per week.

The exclusion requirements were the presence of DOMS, no medical complaints (pain, illness, and/or injury) in the last 6 months that would interfere with testing, and that they were not available for a 1-year follow-up period. Pain and complaints localized below the shoulders and above the lower gluteal folds, with or without pain in the legs (i.e., sciatica) were defined as LBP [41]. Players who were absent from training and competitions for more than three days due to non-contact LBP (minor injury defined as 4 to 7 days without training or competition) were assigned to the non-contact LBP group [42]. The players were advised by the National Technical Plan’s medical team. In addition, players who did not complete the entire ROM-SPORT I battery or survey were not included in the statistical analysis.

### 2.4. Procedure Investigation Research

#### 2.4.1. Survey

As part of the National Technical Plan, players provided information on age, body composition, IH antecedents (year of experience; exposure to training was evaluated as training months per year, training days and hours per week, and exposure to competition), competition level, and non-contact LBP antecedents.

For anthropometry, the measurement standards used were those approved by ISAK. An electronic column scale with stadiometer Seca 799 (Hamburg, Germany) was used to measure mass and height with a precision of 0.5 kg and 0.1 cm, respectively. A correction of 0.5 kg was made for the weight of the clothing. The BMI was considered as body mass divided by height squared (kg/m^2^).

The non-contact LBP history was recorded 1 year after the end of the National Technical Plan. The aim of this part of the survey was to detect IH players with non-contact LBP who would subsequently form the effect (non-contact LBP group or outcome cohort). If the participant had complaints and pain within the last year that led to absenteeism from training and competition of more than three days [yes or no (minor or higher severity)], he was included in the non-contact LBP group [42].

#### 2.4.2. Assessment Procedure ROM-SPORT I Battery

Maximum and passive ROM for 11 movements of the lower extremity were measured for the dominant and non-dominant leg of each IH player using the procedure ROM-SPORT I battery [43].

The evaluators measured ROM, hip extension with the knee relaxed (HE), hip external rotation (HER), and hip internal rotation (HIR) with the knee flexed, flexion with the knee flexed (HF-KF) and extended (HF-KE), adduction with the hip flexed (HAD-HF), abduction with the hip flexed (HAB-HF) and neutral hip/knee (HAB), knee flexion (KF) and ankle dorsiflexion with the knee flexed (AD-KF), and extended (AD-KE).

The ROM was assessed with an inclinometer (ISOMED Unilevel, Inc., Portland, OR, USA) based on inclinometer techniques [43], with the exception of abduction ROM (HAB), which was measured with a 360° stainless steel goniometer (Baseline^®^ Stain-Less, Fabrication Enterprises Inc., New York, NY, USA). Previous research has shown that the reliability of the methods used by the evaluators is moderate to high (coefficients of variation between 0.2% and 9.1%).

### 2.5. Statistical Analysis

The sample size required for this investigation research was previously determined a priori by using the G*Power software package (version 3.1.9.4, University of Düsseldorf, Düsseldorf, Germany) to control an appropriate power (1-ß error probability). The effect size (effect size = 1.05) was determined from the significant difference in lower-extremity ROM (HER and HIR) between the group without non-contact LBP and the asymptomatic group in a sample of IH players [15].

The open-source statistical software used for the data processing was Jamovi 1.6.23 (https://www.jamovi.org (accessed on 5 August 2024)). The Shapiro–Wilk test is used for testing normality. Abnormally distributed data showed a Gaussian distribution after the log transformation. The descriptive statistics were reported as mean and standard deviation and with a 95% CI for the continuous variables of the participant characteristics.

An ANOVA analysis was previously performed to control for random effects, with the risk factors included as dependent variables and the dichotomous variable (non-contact LBP group versus asymptomatic group) as fixed factors. The Bayesian Student’s *t*-test calculated the differences in ROM between the dominant and non-dominant sides for hips, knees, and ankles. The Bayesian Student’s *t*-test for independent samples was calculated for the differences in the mean values between the non-contact LBP group and the asymptomatic group. The BF_10_ was then categorized according to the interpretative cut-offs established by Lee and Wagenmakers [44]. The categories represent the strength of evidence supporting the alternative hypothesis (H1): anecdotal evidence (BF_10_ = 1 to 3), moderate evidence (BF_10_ = 3 to 10), strong evidence (BF_10_ = 10 to 30), very strong evidence (BF_10_ = 30 to 100) and extreme evidence (BF_10_ > 100). Moderate evidence (BF_10_ > 3) was determined sufficiently robust to describe the main effects [44]. For between-group comparisons, the mean and 95% interval of the credible posterior distribution of the standardized effect size (δ) were calculated.

The risk factors (anthropometry, age, hockey experience, training/competition exposure, lower-extremity ROM) associated with non-contact LBP were examined by binary logistic regression analysis with the Enter method. For this purpose, the odds ratio (OR) or odds ratios, the sign of the estimate, the standard error, the z, the *p*-value, and the associated 95% confidence intervals (CI) were determined. The effect sizes for the OR were then categorized according to the interpretative limits set by Batterham and Hopkins [45].

The optimal cut-off value of the risk factors was calculated using a ROC (receiver operating characteristic) analysis. The area under the curve (AUC) determined the predictive ability of the predictors for non-contact LBP. The AUC was then categorized according to the interpretative cut-offs established by Hosmer, Lemeshow, and Sturdivant [46]. The optimal cut-off value for the risk factors, or the value that provided the best discriminatory ability between asymptomatic players and players with non-contact LBP, was then determined using the Youden index. The positive predictive value (PPV), i.e., the probability that a participant with a positive test (limited ROM) has non-contact LBP, and the negative predictive value (NPV), i.e., the probability that a participant with a negative test (normal ROM) does not have non-contact LBP, were determined.

Bayesian contingency table tests (BF_10_ and log odds ratio, 95% credible interval) were used to calculate the correlation between the identified factors (non-contact LBP high risk for the optimal cut-off value) and non-contact LBP.

## 3. Results

A minimum sample of 40 IH players (effect size = 1.05) is needed to have a power of 0.955. Finally, forty-nine IH players met the predefined inclusion and exclusion criteria. Sixteen players (32.7%) experienced a non-contact LBP (minor injury) during the 1-year surveillance period.

Differences in HF-KE ROM were found by ANOVA (random effects) analysis (F_1,47_ = 29.539; *p* < 0.001; η^2^ = 0.386; 8.26°). Lateral differences were found in hip ROM (HAD-HF: 25.0° vs. 26.7°, BF_10_ = 83.5, 95% credible interval = −0.52 [−0.82, −0.23], very strong evidence; HAB-KF: 37.6° vs. 35.8°, BF_10_ = 1169.0, 95% credible interval = 0.65 [0.34, 0.96], very strong evidence). The individual analysis showed no asymmetry of more than 6° in either test. Therefore, the mean value of both sides was used for further statistical analysis.

Differences between the non-contact LBP players and the asymptomatic players (Table 1) were found in HF-KE ROM (62.3° vs. 70.5°; BF_10_ = 6984.0, 95% credible interval = 1.53 [0.83, 2.23], extreme evidence). The group of IH players with non-contact LBP showed a reduced ROM of the HF-KE ROM of 8.2° (mean value between the two groups).

The Enter method showed an association between the non-contact LBP and the HF-KE ROM for H1 (χ^2^(39) = 43.939; *p* < 0.001). The Bayesian Information Criteria and Akaike Information Criteria for the model fit were 56.885 and 37.967, respectively. The R^2^ showed good model fit, 75.0% for McFadden’s R^2^, 61.2% for Cox and Snell´s R^2^, and 85.4% for Nagelkerke´s R^2^. The HF-KE ROM (*p* = 0.015; OR = 3.850 [large]; 95% CI = 1.293 to 11.463) was the predictor variable in the IH players. The performance diagnostics of the model showed a sensitivity of 0.938 (15 out of 16) and a specificity of 1.000 (33 out of 33).

The ROC curves showed good predictive precision of the model for non-contact LBP, which was statistically significant (*p* = 0.000; AUC = 0.941; standard error: 0.03; 95% CI: 0.87 to 1.00). The HF-KE ROM cut-off value that most accurately identified individuals at risk of causing non-contact LBP was set at 67° (sensitivity = 93.94%, specificity = 81.25%; Youden Index = 0.752). The likelihood that a player with a value of 67° or less would suffer from non-contact LBP was 91.18% (positive predictive value), and the probability that a player with a value greater than 67° would suffer from non-contact LBP was 86.67% (negative predictive value).

The 70% (14/20) of IH players with an HF-KE ROM of 67° or less will cause a non-contact LBP in the future (BF_10_ = 18404.83 [strong]; log odds ratio = 3.192 [1.614 to 4.769]).

## 4. Discussion

Low back pain is a very common symptom in children and older IH players [3]. HER and total hip rotation are the movements with the strongest association with non-contact LBP [15]. However, in the present research, among all risk factors assessed (age, body composition, IH antecedents, competition level, and hip, knee, and ankle ROM), the variable most strongly associated with non-contact LBP was limited HF-KE ROM (hamstring tightness). Hamstring tightness has also been associated with non-contact LBP in other sports such as football [31], basketball [31], baseball [32], and skiing [47]. In youth IH players, hamstring tightness was found in 100% of athletes [21].

Hamstring tightness leads to compensatory movements in the alignment of the sagittal spine-pelvis-leg alignment [48] and alters the sequence of movements of the lumbar-pelvic rhythm, especially in the trunk forward flexion position [49]. In IH, players adopt the trunk-bent position as the usual or base position [50]. Tightness in the hamstrings limits the anteversion of the pelvis and the maintenance of normal lumbar lordosis [48] during technical-tactical situations in IH. Retroversion of the pelvis causes inversion of the lumbar curve and thoracic hyperkyphosis. In the lumbar region, compression of the intervertebral discs occurs in the anterior part of the fibrous ring, causing posterior migration of the nucleus [51]. This can lead to damage to the discs [52] and increased tension in the passive elements of the posterior part of the spine, such as the posterior longitudinal, intertransverse, interspinous, and supraspinous ligaments; the zygapophyseal joint capsule; and the facet joint capsule [53]. Under these conditions, there is an increased risk of non-contact LBP [54,55]. Sagittal lumbar deformities cause spinal pathologies [56,57,58,59] and non-contact LBP in sports such as hockey [57], soccer [56,58,59], and basketball [58] players.

Trunk flexion, the basic position in IH players, is considered one of the postures with the highest intradiscal pressure [53,60]. It is possible that this mechanism is the reason for the 32.7% prevalence of non-contact LBP in IH players in this research. These findings suggest that the physical-technical demands on the sagittal spine-pelvic-leg plane during regular training and the basic position in IH may be an important consideration in determining which impairments are most likely to be investigated and considered in prevention and intervention strategies. To reduce the risk of non-contact LBP, IH players require optimal hamstring flexibility to allow for proper sagittal alignment of the pelvis and spine in the sport’s base position of trunk flexion. Specifically, IH players should learn optimal reference values for the lower-limb flexibility profile in IH, with particular attention to hip rotation (external and internal) and HF-KE ROM. The achievement of these quantitative goals must be complemented by qualitative goals related to the effectiveness and safety of the flexibility program. Optimal ROM of hip rotation (especially internal and total hip rotation) should also be achieved to minimize compensatory movements of the pelvis in the transverse plane [15,29]. Therefore, we suggest including in the IH player’s training program the static-active stretching technique that allows anteversion of the pelvis prior to trunk flexion to ensure proper sagittal spine alignment and safety during the hamstring stretch [61,62]. In addition, players benefit from the intermuscular coordination between agonists (activation of the anterior pelvic muscles) and antagonists (relaxation of the pelvic retroversion) [61,62]. In addition, this technique can be complemented by the technique of eccentric stretching, which allows the hamstrings to maintain the muscular properties required for eccentric actions during the game of IH [50]. Finally, flexibility training should be complemented by strengthening the spine and hip muscles and improving sagittal and transverse posture.

Another possible biomechanical alternative is to bend the knees more and bend the trunk less in order to avoid the restriction of the hamstring muscles in players with shortness. This biomechanical alternative avoids the retroversion of the pelvis and the resulting sagittal misalignments of the spine, especially in the release phase (beginning of the impulse phase where the body bends forward to accelerate the movement) and the follow-through phase (full impulse phase with full extension of the hip, knee, and plantar flexion of the ankle) of basic IH techniques such as starts (rapid acceleration from a stopped position) and linear forward skating [63,64].

As far as the authors know, this is the first research to determine a diagnostic cut-off for ROM high risk for developing non-contact LBP in 8- to 15-year IH players. The criterion cut-off (or predictive value) for the occurrence of non-contact LBP was set at 67°. In children’s IH players [21], the reference value for the criterion to identify the risk of non-contact LBP due to limited HF-KE ROM (hamstring tightness) was set at 88°, which was determined in senior football players. As flexibility depends on the particular movement, sex, age, and level of competition [43], 88° is not considered an appropriate cut-off for youth IH players. Other studies that have evaluated HF-KE ROM in athletes with non-contact LBP have obtained a higher threshold value than the one obtained in this research (62.2°). In 20 IH players aged 18–29 years, 72° was found for HF-KE ROM [37], 71° in 94 senior football and basketball players [31], and 70° in 335 adolescent baseball players [32]. In 74 children’s IH players [21], hamstring tightness was defined as 66.3°.

Skating is the most important skill, representing a specific movement pattern that is very different from the collective sports that require running (hockey, basketball, handball, football, or futsal). The skating stride requires a high degree of plantar flexion and dorsiflexion of the ankles, knee extension and flexion, hip extension and flexion, hip adduction and abduction, and external and internal rotation of the hip. The skating stride pattern requires hip flexion, extension, and abduction in a trunk flexion position [50]. Trunk flexion requires optimal hamstring extensibility to promote pelvic anteversion and normal sagittal alignment of the lumbar spine. It is possible that players with the lowest HF-KE ROM values (range in the current research: 56°–86°) perform the basic game actions with retroversion of the pelvis and kyphosis of the lumbar spine, resulting in an overload of the lumbar tissues [51,53,60] and LBP [54,55].

To summarize, the goal of IH players is to reach more than 67° (HF-KE ROM) in order to reduce the risk factor related to flexibility and improve the technical posture in this sport. The inclusion of flexibility training helps to reach the target values of HF-KE ROM training at 67° [25,65,66]. It should be considered that the occurrence of LBP can be prevented if flexibility training (stretching of the hamstring and hip rotator muscles) in IH players is complemented by lumbar flexibility, core strengthening, and correcting posture [26,27,28]. However, this type of physical intervention can be useful and safe in IH players with non-contact LBP associated with a pathology of the lumbar spine (hernia of the nucleus pulposus, osteoarthritis, thickening of the ligamentum flavum, etc.), i.e., when the etiology is mechanical (faulty lumbopelvic or pelvifemoral rhythm) and bilateral leg pain is detected on physical examination [67].

### Future Studies and Limitations

Future studies should replicate the method with a larger participation of IH players taking part in the National Technical Plan, assess other risk factors (agility, balance, trunk stability, leg stiffness, and change of direction tests), and extend the follow-up time of players. With this in mind, the risk of non-contact LBP could be better predicted by increasing the number of participants with non-contact LBP. Regarding dropout rates, a greater understanding by National Technical Plan directors of the importance of assessing risk factors for non-contact LBP (e.g., publication of this research work) will help to ensure that investigators have more time to assess all participants in future studies. One limitation is the use of statistical approaches (logistic regression), which, unlike certain supervised learning algorithms (i.e., ensemble, class balance, and cost-sensitive learning techniques), have not been specifically developed to deal with class imbalance problems. The number of injured (or symptomatic) players (minority class) prospectively reported is always much lower than the healthy (or asymptomatic) players (majority class), as observed in this study (non-contact LBP group [*n* = 16] versus asymptomatic group [*n* = 33]). Thus, in this scenario, traditional logistic regression is often biased towards the majority class (asymptomatic group), and therefore, there is a higher misclassification rate for the minority class instances (non-contact LBP group). In addition, this study lacks a correction for multiple comparisons between the groups.

Also, binary logistic regression analysis is observed that usually has a high specificity (true negatives) but a very low sensitivity (true positives). Therefore, the use of a more comprehensive and accurate statistical and conceptual approach, such as machine learning, is required. Although the outcome cohort (non-contact LBP group) was conducted according to the definition and severity (absence from training and competitions), the subjectivity of the questionnaire and the heterogeneity of training (type and load) may also influence the risk of non-contact LBP. In addition, the increasing professionalization of this new sport could show different results in the coming years due to the influence of a greater volume and load of annual training and competitions. On the other hand, participants were only tested at the end of the pre-season, while subsequent LBP was monitored throughout the entire season (1 year).

Based on previous reports of hip spine syndrome in IH players with LBP”, HF-KE ROM improvement should be considered. The role of limited HF-KE ROM in the etiology and treatment of LBP in IH players should be the subject of future studies.

## 5. Conclusions

This research shows that the ROM of hip flexion with the knee extended is a risk factor for the cause of non-contact LBP in IH players. In the data examined, a ROM of hip flexion with the knee extended of 67° or less was determined to be the most appropriate cut-off value for prognostic screening for non-contact LBP in IH players aged 8 to 15 years. The identification of modifiable factors, such as flexibility in players with a history of non-contact LBP, could allow the development of more specific and effective pre-season injury prevention programs.

There were limitations regarding the sample size and imbalance of the two cohorts; the subjectivity of the questionnaire and the heterogeneity of training (type and load) may also influence the risk of non-contact LBP.

## Figures and Tables

**Table 1:** Age, body composition, sport antecedents, and range of motion differences of the inline hockey players by low back pain.

Variables		Asymptomatic Group (*n* = 33)	Low Back Pain Group (*n* = 16)	Bayesian Factor and Evidence	δ (95% Credible Interval)
Age and body composition	Age (y) *	11.81 ± 1.77	12.00 ± 0.89	0.31 Anecdotical	−0.09 (−0.63, 0.43)
	Body mass (kg)	48.58 ± 11.55	52.18 ± 5.62	0.52 Anecdotical	−0.28 (−0.85, 0.24)
	Body height (cm)	152.27 ± 11.22	155.12 ± 7.76	0.41 Anecdotical	−0.21 (−0.77, 0.30)
	Body mass index (kg/m^2^)	20.67 ± 3.01	21.85 ± 3.34	0.55 Anecdotical	0.29 (−0.86, 0.23)
Antecedents	Experience (y) *	3.24 ± 1.45	2.87 ± 1.82	0.37 Anecdotical	0.18 (−0.34, 0.73)
	Months per year *	9.75 ± 0.93	10.25 ± 0.44	1.42 Anecdotical	−0.49 (−1.09, 0.05)
	Days per week *	2.93 ± 0.24	2.75 ± 0.44	1.30 Anecdotical	0.48 (−0.07, 1.07)
	Hours per week *	4.43 ± 1.42	3.75 ± 0.93	1.01 Anecdotical	0.43 (−0.11, 1.02)
Range of motion (degree)	HE	11.30 ± 5.61	10.25 ± 4.40	0.35 Anecdotical	0.15 (−0.37, 0.70)
	HAD-HF *	25.93 ± 3.65	25.87 ± 5.30	0.30 Anecdotical	0.01 (−0.52, 0.54)
	HAB	36.90 ± 2.89	36.37 ± 2.91	0.34 Anecdotical	0.14 (−0.38, 0.69)
	HIR *	47.57 ± 6.86	47.25 ± 10.80	0.30 Anecdotical	0.03 (−0.50, 0.56)
	HER *	60.97 ± 7.54	61.37 ± 5.53	0.30 Anecdotical	−0.04 (−0.58, 0.48)
	HAB-HF	65.09 ± 7.37	62.62 ± 5.16	0.53 Anecdotical	0.28 (−0.24, 0.85)
	HF-KE *	**70.51 ± 5.52**	**62.25 ± 3.58**	**6980.00** **Extreme**	**1.53** **(0.83, 2.23)**
	HF-KF	134.57 ± 6.50	136.37 ± 5.21	0.43 Anecdotical	−0.23 (−0.79, 0.29)
	KF	120.18 ± 9.16	118.00 ± 6.47	0.40 Anecdotical	0.40 (−0.32, 0.76)
	AD-KE	34.27 ± 4.99	33.37 ± 3.30	0.35 Anecdotical	0.15 (−0.37, 0.70)
	AD-KF	39.24 ± 6.03	40.00 ± 4.89	0.32 Anecdotical	−0.10 (−0.64, 0.42)

* variables that changed in logarithmic transformation. HE: hip extension with the knee relaxed; HAD-HF: hip adduction with the hip flexed; HAB: hip abduction with the neutral hip/knee flexed; HIR: hip internal rotation with the knee flexed; HER: hip external rotation with the knee flexed; HAB-HF: hip abduction with the hip flexed; HF-KE: hip flexion with the knee extended; HF-KF: hip flexion with the knee flexed; KF: knee flexion; AD-KE: ankle dorsiflexion with the knee extended; AD-KF: ankle dorsiflexion with the knee flexed.

## Data Availability

Updated databases and codes are freely available in Mendeley Data (https://data.mendeley.com/datasets/ggyknprwz7/1 (accessed on 28 October 2023)).

## Supplementary Materials

The following supporting information can be downloaded at: https://www.mdpi.com/article/10.3390/children11121517/s1, File S1: TRIPOD checklist.

## References

[1] Hutchinson M., Milhouse C., Gapski M. (1988). Comparison of injury patterns in elite hockey players using ice versus in-line skates. Med. Sci. Sports Exerc..

[2] Varlotta G., Lager S., Nicholas S., Browne M., Schlifstein T. (2000). Professional roller hockey injuries. Clin. J. Sport Med..

[3] Moreno-Alcaraz V., Cejudo A., Sainz de Baranda P. (2020). Injury types and frequency in Spanish inline hockey players. Phys. Ther. Sport.

[4] Rejeb A., Johnson A., Farooq A., Verrelst R., Pullinger S., Vaeyens R., Witvrouw E. (2019). Sports injuries aligned to predicted mature height in highly trained Middle-Eastern youth athletes: A cohort study. BMJ Open.

[5] Bult H., Barendrecht M., Tak I. (2018). Injury Risk and Injury Burden Are Related to Age Group and Peak Height Velocity Among Talented Male Youth Soccer Players. Orthop. J. Sports Med..

[6] Hawkins D., Metheny J. (2001). Overuse injuries in youth sports: Biomechanical considerations. Med. Sci. Sports Exerc..

[7] Vaeyens R., Malina R., Janssens M., Van Renterghem B., Bourgois J., Vrijens J., Philippaerts R. (2006). A multidisciplinary selection model for youth soccer: The Ghent Youth Soccer Project. Br. J. Sports Med..

[8] Van Hilst J., Hilgersom N., Kuilman M., Paul P., Kuijer F.M., Frings-Dresen M. (2015). Low back pain in young elite field hockey players, football players and speed skaters: Prevalence and risk factors. J. Back Musculoskelet. Rehabil..

[9] Cugusi L., Manca A., Fischbach E., Secci C., Bergamin M., Gobbo S., Di Blasio A., Montella A., Bandiera P., Deriu F. (2021). Low back pain prevalence and risk factors in Italian adolescent male soccer players: Results from an online survey. J. Sports Med. Phys. Fit..

[10] Makovicka J., Deckey D., Patel K., Hassebrock J., Chung A., Tummala S., Hydrick T., Pena A., Chhabra A. (2019). Epidemiology of Lumbar Spine Injuries in Men’s and Women’s National Collegiate Athletic Association Basketball Athletes. Orthop. J. Sports Med..

[11] Bere T., Alonso J., Wangensteen A., Bakken A., Eirale C., Dijkstra H., Ahmed H., Bahr R., Popovic N. (2015). Injury and illness surveillance during the 24th Men’s Handball World Championship 2015 in Qatar. Br. J. Sports Med..

[12] Trompeter K., Fett D., Platen P. (2017). Prevalence of Back Pain in Sports: A Systematic Review of the Literature. Sports Med..

[13] Post E., Simon J., Robison H., Morris S., Bell D. (2022). Epidemiology of overuse injuries in US secondary school athletics from 2014–2015 to 2018–2019 using the national athletic treatment, injury and outcomes network. J. Athl. Train..

[14] Rees H., Delahunt E., Boreham C., Blake C. (2020). Epidemiology of injuries in senior men’s field hockey: A two-season prospective observational injury surveillance study. J. Sports Sci..

[15] Cejudo A., Moreno-Alcaraz V., Izzo R., Santonja-Medina F., Sainz de Baranda P. (2020). External and Total Hip Rotation Ranges of Motion Predispose to Low Back Pain in Elite Spanish Inline Hockey Players. Int. J. Environ. Res. Public Health.

[16] Alricsson M., Björklund G., Cronholm M., Olsson O., Viklund P., Svantesson U. (2016). Spinal alignment, mobility of the hip and thoracic spine and prevalence of low back pain in young elite cross-country skiers. J. Exerc. Rehabil..

[17] Ohlén G., Wredmark T., Spangfort E. (1989). Spinal sagittal configuration and mobility related to low-back pain in the female gymnast. Spine.

[18] Cejudo A., Centenera-Centenera J., Santonja-Medina F. (2021). Sagittal Integral Morphotype of Competitive Amateur Athletes and Its Potential Relation with Recurrent Low Back Pain. Int. J. Environ. Res. Public Health.

[19] Baranto A., Hellström M., Cederlund C., Nyman R., Swärd L. (2009). Back pain and MRI changes in the thoraco-lumbar spine of top athletes in four different sports: A 15-year follow-up study. Knee Surg. Sports Traumatol. Arthrosc..

[20] Sainz de Baranda P., Cejudo A., Moreno-Alcaraz V., Martinez-Romero M., Aparicio-Sarmiento A., Santonja F. (2020). Sagittal spinal morphotype assessment in 8 to 15 years old Inline Hockey players. PeerJ.

[21] Cejudo A., Moreno-Alcaraz V., De Ste Croix M., Santonja-Medina F., Sainz de Baranda P. (2020). Lower-Limb Flexibility Profile Analysis in Youth Competitive Inline Hockey Players. Int. J. Environ. Res. Public Health.

[22] Leppänen M., Aaltonen S., Parkkari J., Heinonen A., Kujala U. (2014). Interventions to prevent sports related injuries: A systematic review and meta-analysis of randomised controlled trials. Sports Med..

[23] Ayala F., Robles-Palazón F., Blázquez-Rincón D., López-Valenciano A., López-López J., De Ste Croix M. (2024). A systematic review and network meta-analysis on the effectiveness of exercise-based interventions for reducing the injury incidence in youth team-sport players. Part 2: An analysis by movement patterns. Ann. Med..

[24] Robles-Palazón F., Blázquez-Rincón D., López-Valenciano A., Comfort P., López-López J., Ayala F. (2024). A systematic review and network meta-analysis on the effectiveness of exercise-based interventions for reducing the injury incidence in youth team-sport players. Part 1: An analysis by classical training components. Ann. Med..

[25] Sugiura Y., Sakuma K., Sakuraba K., Sato Y. (2017). Prevention of Hamstring Injuries in Collegiate Sprinters. Orthop. J. Sports Med..

[26] Teferi G. (2020). Regular Physical Exercise for Prevention and Treatment of Low Back Pain: A Systematic Review. Am. J. Sports Sci. Med..

[27] Jones M., Stratton G., Reilly T., Unnithan V. (2007). Recurrent non-specific low-back pain in adolescents: The role of exercise. Ergonomics.

[28] Jones M., Stratton G., Reilly T. (2007). The efficacy of exercise as an intervention to treat recurrent nonspecific low back pain in adolescents. Pediatr. Exerc. Sci..

[29] Van Dillen L., Bloom N., Gombatto S., Susco T. (2008). Hip rotation range of motion in people with and without low back pain who participate in rotation-related sports. Phys. Ther. Sport.

[30] Cejudo A., Ginés-Díaz A., Sainz de Baranda P. (2020). Asymmetry and Tightness of Lower Limb Muscles in Equestrian Athletes: Are They Predictors for Back Pain?. Symmetry.

[31] Cejudo A., Centenera-Centenera J., Santonja-Medina F. (2021). The Potential Role of Hamstring Extensibility on Sagittal Pelvic Tilt, Sagittal Spinal Curves and Recurrent Low Back Pain in Team Sports Players: A Gender Perspective Analysis. Int. J. Environ. Res. Public Health.

[32] Kato K., Otoshi K., Tominaga R., Kaga T., Otoshi K., Igari T., Sato R., Konno S. (2022). Influences of limited flexibility of the lower extremities and occurrence of low back pain in adolescent baseball players: A prospective cohort study. J. Orthop. Sci..

[33] Vad V., Bhat A., Basrai D., Gebeh A., Aspergren D., Andrews J. (2004). Low Back Pain in Professional Golfers: The Role of Associated Hip and Low Back Range-of-Motion Deficits. Am. J. Sports Med..

[34] Bahr R. (2016). Why screening tests to predict injury do not work—And probably never will: A critical review. Br. J. Sports Med..

[35] Talari K., Goyal M. (2020). Retrospective studies—Utility and caveats. J. R. Coll. Physicians Edinb..

[36] Collins G., Reitsma J., Altman D., Moons K. (2015). Transparent Reporting of a Multivariable Prediction Model for Individual Prognosis or Diagnosis (TRIPOD). Circulation.

[37] Cejudo A., Moreno-Alcaraz V., Izzo R., Robles-Palazón F., Sainz de Baranda P., Santonja-Medina F. (2020). Flexibility in Spanish Elite Inline Hockey Players: Profile, Sex, Tightness and Asymmetry. Int. J. Environ. Res. Public Health.

[38] Vescovi J., Murray T., VanHeest J. (2006). Positional performance profiling of elite ice hockey players. Int. J. Sports Physiol. Perform..

[39] Bandyopadhyay A., Datta G., Dey S. (2019). Body composition characteristics and physiological performance tests of junior elite field hockey players according to different playing positions. J. Phys. Educ. Sport.

[40] Ferraz A., Valente-Dos-santos J., Sarmento H., Duarte-Mendes P., Travassos B. (2020). A Review of Players’ Characterization and Game Performance on Male Rink-Hockey. Int. J. Environ. Res. Public Health.

[41] Foss I., Holme I., Bahr R. (2012). The Prevalence of Low Back Pain Among Former Elite Cross-Country Skiers, Rowers, Orienteerers, and Nonathletes A 10-Year Cohort Study. Am. J. Sports Med..

[42] (2020). International Olympic Committee Injury and Illness Epidemiology Consensus Group International Olympic Committee consensus statement: Methods for Recording and Reporting of Epidemiological Data on Injury and Illness in Sports 2020 (Including the STROBE Extension for Sports Injury and Illness Surveillance (STROBE-SIIS)). Orthop. J. Sports Med..

[43] Cejudo A. (2022). Description of ROM-SPORT I Battery: Keys to Assess Lower Limb Flexibility. Int. J. Environ. Res. Public Health.

[44] Lee M., Wagenmakers E. (2013). Bayesian Data Analysis for Cognitive Science: A Practical Course.

[45] Batterham A., Hopkins W. (2006). Making meaningful inferences about magnitudes. Int. J. Sports Physiol. Perform..

[46] Hosmer D., Lemeshow S., Sturdivant R. (2013). Applied Logistic Regression.

[47] Noormohammadpour P., Rostami M., Ali Mansournia M., Farahbakhsh F., Hosein Pourgharib Shahi M., Kordi R. (2016). Low back pain status of female university students in relation to different sport activities. Eur. Spine J..

[48] Fasuyi F., Fabunmi A., Adegoke B. (2017). Hamstring muscle length and pelvic tilt range among individuals with and without low back pain. J. Bodyw. Mov. Ther..

[49] Harris-Hayes M., Sahrmann S., Van Dillen L. (2009). Relationship between the hip and low back pain in athletes who participate in rotation-related sports. J. Sport Rehabil..

[50] Shell J., Robbins S., Dixon P., Renaud P., Turcotte R., Wu T., Pearsall D. (2017). Skating start propulsion: Three-dimensional kinematic analysis of elite male and female ice hockey players. Sports Biomech..

[51] Alexander L., Hancock E., Agouris I., Smith F. (2007). The response of the nucleus pulposus of the lumbar intervertebral discs to functionally loaded positions. Spine.

[52] Castanharo R., Duarte M., McGill S. (2014). Corrective sitting strategies: An examination of muscle activity and spine loading. J. Electromyogr. Kinesiol..

[53] Wilke H., Neef P., Hinz B., Seidel H., Claes L. (2001). Intradiscal pressure together with anthropometric data–a data set for the validation of models. Clin. Biomech..

[54] Reis F., Macedo A. (2015). Influence of Hamstring Tightness in Pelvic, Lumbar and Trunk Range of Motion in Low Back Pain and Asymptomatic Volunteers during Forward Bending. Asian Spine J..

[55] Hasebe K., Sairyo K., Hada Y., Dezawa A. (2014). Spino-pelvic-rhythm with forward trunk bending in normal subjects without low back pain. Eur. J. Orthop. Surg. Traumatol..

[56] Wodecki P., Guigui P., Hanotel M., Cardinne L., Deburge A. (2002). Sagittal alignment of the spine: Comparison between soccer players and subjects without sports activities. Rev. Chir. Orthop. Reparatrice l’Appareil Mot..

[57] Sivanich A., Shultz J., Raja A. (2018). Chronic Lumbar Spondylolysis in an Adolescent Hockey Player. JBJS J. Orthop. Physician Assist..

[58] Lawrence K., Elser T., Stromberg R. (2016). Lumbar spondylolysis in the adolescent athlete. Phys. Ther. Sport.

[59] Purcell L., Micheli L. (2009). Low back pain in young athletes. Sports Health.

[60] Nachemson A. (1981). Disc pressure measurements. Spine.

[61] Ayala F., Sainz de Baranda P., Cejudo A. (2012). El entrenamiento de la flexibilidad: Técnicas de estiramiento. Rev. Andal. Med. Deporte.

[62] Sullivan M., Dejulia J., Worrell T. (1992). Effect of pelvic position and stretching method on hamstring muscle flexibility. Med. Sci. Sports Exerc..

[63] Minkoff J., Varlotta G., Simonson B. (1994). Ice Hockey.

[64] Moreno Alcaraz V. (2019). Lesiones, Morfotipo Sagital de la Columna Vertebral, Perfil de Flexibilidad de la Extremidad Inferior y Dolor de Espalda en Jugadores de Hockey Linea.

[65] Moreira R., Akagi F., Wun P., Moriguchi C., Sato T. (2012). Effects of a school based exercise program on children’s resistance and flexibility. Work.

[66] Palmer T., Thiele R. (2019). Passive stiffness and maximal and explosive strength responses after an acute bout of constant-tension stretching. J. Athl. Train..

[67] Foreman M., Maddy K., Patel A., Reddy A., Costello M., Lucke-Wold B. (2023). Differentiating Lumbar Spinal Etiology from Peripheral Plexopathies. Biomedicines.

